# Retraction: Muscle-derived stem cell exosomes with overexpressed miR-214 promote the regeneration and repair of rat sciatic nerve after crush injury to activate the JAK2/STAT3 pathway by targeting PTEN

**DOI:** 10.3389/fnmol.2026.2021959

**Published:** 2026-10-16

**Authors:** Frontiers Editorial Office

The journal retracts the 26 May 2023 article cited above.

Following publication, concerns were raised regarding the integrity of the images in the published figures. Image duplication concerns were identified in Figures 3B and 4D. Upon thorough evaluation, it was determined that the article requires substantial revisions that go beyond the scope of a standard correction. Therefore, the decision has been made to retract the current manuscript to allow for a comprehensive revision and resubmission.

This retraction was approved by the Chief Editors of Frontiers in Molecular Neuroscience and the Chief Executive Editor of Frontiers. The authors have not responded to correspondence regarding this retraction.

